# 

**Published:** 1913-10-15

**Authors:** 


### ANNOUNCEMENTS.

### FALL MEETING OF EXAMINING BOARDS.

Michigan Board, meets at Ann Arbor, November 10th to 15th.

Dr. F. E. Sharp, Secy.,
Pt. Huron, Mich.

---

Indiana Board, meets at the State House in Indianapolis, November 10th to 15th.

F. R. Henshaw, Secy.
K. of P. Building,
Indianapolis.

---

Massachusetts Board, will meet in Boston, October 22nd to 24th.

Dr. G. E. Mitchell, Secy.
14 Water St., Haverhill,
Mass.

---

Maryland Board, meets November 6th and 7th, at Baltimore College of Dental Surgery, Baltimore.

Dr. F. F. Drew, Secy,.
701 Howard St., Baltimore,
Md.

---

Connecticut Board, will meet in Hartford, November 13th to 15th.

Edward Eberle, Secy.,
902 Main St., Hartford, Conn.

ILLINOIS BOARD, will meet in Chicago, at University of Illinois Dental Building, Honore and Harrison Sts. November 10th, 1913.

DR. O. H. SEIFERT, Secy,
Ridgely Bank Bldg.,
Springfield, Ills.

---

### THE PANAMA-PACIFIC DENTAL CONGRESS.

The work of the Committe of Organization of the Panama-Pacific Dental Congress is rapidly assuming definite form, and the entire general plan of the Congress will shortly be announced.

The floor plans of the new Municipal Auditorium, in which the Congress will meet, will be sent to all prospective exhibitors within the next thirty days. The exhibits will be held in the main hall of the Auditorium, a room 190 feet square, affording ample space and light, and from present indications, all of this great area will be fully occupied. It is planned to make these exibits, and their accompanying clinics, one of the great features of the Congress, and they will, aside from the general program, afford a liberal education to any one interested in modern dentistry.

Space in the Auditorium has been reserved for the general sessions of the Congress, and for the meetings of it's sections, and also for the dental societies and fraternities which will meet in San Francisco during the Congress.

Three hundred thousand gum stickers bearing the seal and date of the Congress, will shortly be placed in the hands of dental dealers throughout the country, and every dentist who receives goods or letters from them, will in this way be reminded that it is time to prepare for a trip to San Francisco in August of 1915, to attend the Panama-Pacific Dental Congress, and the Panama-Pacific International Exposition.

